# Comparison of laparoscopic and robotic surgery of choledochal cyst in pediatrics: single center experience

**DOI:** 10.1007/s00464-025-12222-1

**Published:** 2025-10-23

**Authors:** Jiyong Jang, Dayoung Ko, Joong Kee Youn, Hee-Beom Yang, Hyun-Young Kim

**Affiliations:** 1 Division of Pediatric Surgery, Seoul National University Hospital, Seoul, Korea; 2 Department of Surgery, Seoul National University Bundang Hospital, Seongnam, Korea; 3 Department of Surgery, College of Medicine, Seoul National University, 101 Daehak-ro, Jongno-gu, Seoul, Korea

**Keywords:** Pediatric, Choledochal cyst excision, Laparoscopic procedures, Robot-assisted procedures

## Abstract

**Background:**

Recently, laparoscopic and robot-assisted procedures have become increasingly common for treating pediatric choledochal cysts. This retrospective study aims to evaluate and compare the safety and efficacy of these procedures in pediatric choledochal cyst cases.

**Methods:**

Between April 2017 and September 2023, 60 patients were enrolled in this study; laparoscopic procedures were applied in 45 patients, and robot-assisted procedures in 15. We collected clinical data, including all patients’ demographic information, the cyst’s type and size, and clinical outcomes through a review of medical records. We also conducted a satisfaction survey using validated questionnaires (Glasgow Children’s Benefit Inventory, Gastrointestinal Quality of Life Index) and additional supplementary questions.

**Results:**

For the anastomosis site size (mm), the laparoscopy group (9.38, [5.09;11.55]) showed larger sizes than the robot-assisted group (7.26, [3.68;8.69]) (p = 0.0381). Hospitalization days (d) were longer for the laparoscopy group (14.64, [9;16]) compared to the robot-assisted group (11.13, [8;12]) (p = 0.0224). Although the complications were not statistically significant, the robot-assisted group had no complications. In contrast, the laparoscopy group reported 2 cases of pancreatitis, 1 case of A-loop syndrome, 1 case of chyle ascites, and 1 case of wound complication.

A total of 17 patients responded to the satisfaction survey (28% response rate).

**Conclusion:**

Robot-assisted surgery is a feasible and effective method for treating pediatric choledochal cysts, and its surgical outcomes are comparable with those of laparoscopic procedures.

Choledochal cyst (CC) is a rare disease characterized by an anomaly of the biliary tract. It manifests with symptoms such as abdominal pain and jaundice, which arise from the dilatation of the intrahepatic or extrahepatic bile ducts [1]. If not appropriately treated, CC may lead to complications such as cholangitis, pancreatitis, cyst perforation, and potential progression to cancer [2].

The primary treatment for CC involves completely resecting the cyst via Roux-en-Y hepaticojejunostomy [3]. This procedure was traditionally performed as an open procedure. With the advent of minimally invasive techniques, laparoscopic surgery has gained popularity since its initial application on a 6-year-old girl with a Roux-en-Y hepaticoenterostomy in 1995, reported by Farello et al. [3, 4] Nonetheless, the laparoscopic approach remains technically demanding for hepaticojejunostomy due to the small bile duct diameter and the inherent complications associated with laparoscopic surgery, leading to a steep learning curve [5, 6].

Robotic surgery is also suggested as a viable option for treating CC. Woo et al. [7] first reported a robotic-assisted choledochal cyst resection in children in 2006. Robotic surgery offers several technical advantages as equipment and surgical skills have advanced. Robotic systems, equipped with flexible surgical instruments and a three-dimensional view, enhance surgical precision [8–10]. However, more data from studies comparing laparoscopic and robotic surgery in treating pediatric CC patients needs to be collected. Earlier literature reviewed satisfaction surveys of robot-assisted surgeries across various clinical conditions, indicating a preference for robot-assisted procedures [11–13]. However, there needs to be more satisfaction surveys specifically targeting pediatric choledochal cyst patients treated with robot-assisted surgery.

Since 2020, our organization has adopted robotic surgery, carrying out 15 cases. We also gathered data on 45 cases of laparoscopic surgery. We conducted a retrospective study and an additional satisfaction survey to evaluate and compare the safety and efficacy of laparoscopic and robotic-assisted procedures in pediatric choledochal cyst cases.

## Materials & methods

### Study Population

This retrospective study analyzed patients with choledochal cysts treated with either laparoscopic or robot-assisted procedures using the da Vinci surgical system from April 2017 to September 2023. All procedures were performed at a single center by the same surgeon. Data were collected for patients treated with laparoscopic procedures from April 2017 and those treated with robot-assisted procedures from September 2020. 45 patients were in the laparoscopic group, and 15 were in the robot-assisted group. The study received approval from the Institutional Review Board of Seoul National University Hospital (2210–169-1378).

### Laparoscopic procedures

Laparoscopic surgery was performed using a 5 mm endoeye flex scope (Olympus) after the insertion of 3 or 5 mm trocars in the umbilicus, epigastric region, left side, and right upper quadrant. Following the dissection of the choledochal cyst, the distal common bile duct (CBD) was ligated using a Hem-o-lok surgical clip at the pancreatic border, and the proximal transection was carried out at the level of the common hepatic duct. At this time, a small flexible surgical ruler was inserted to measure the size of the duct at the anastomosis site. Extracorporeal jejunojejunostomy was performed by exteriorizing the intestine through the umbilicus, while laparoscopic hepaticojejunostomy involved interrupted sutures using polyglactin 5–0 or 6–0 threads, depending on the size of the common hepatic duct.

### Robotic procedures

**Fig. 1:**
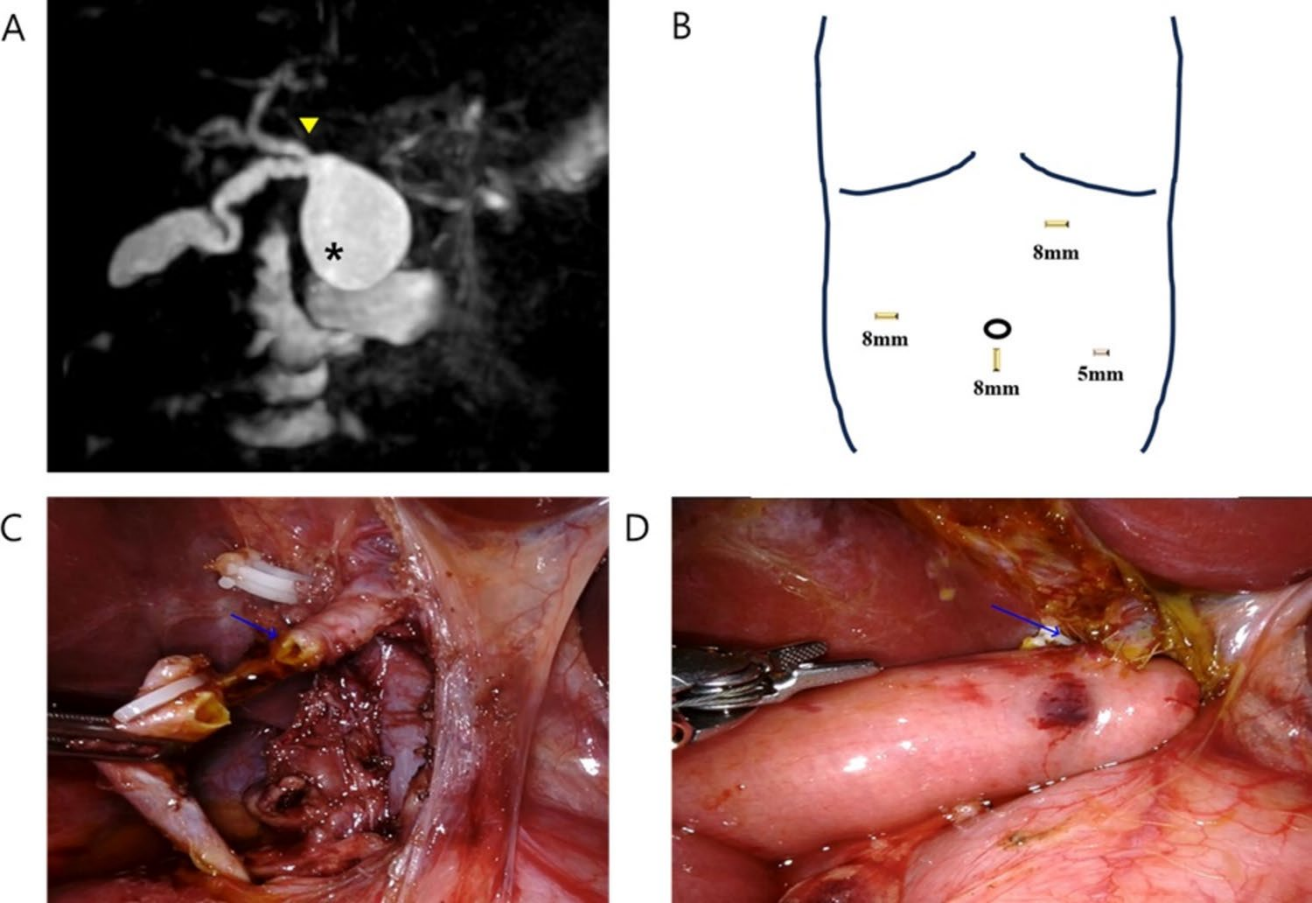
12-month-old female patient underwent robot-assisted choledochal cyst excision: **A** MRCP image of the choledochal cyst. **B** Locations and sizes of the trocars used in the robot-assisted procedure. **C** Intraoperative findings. **D** Hepaticojejunostomy performed intracorporeally

Robotic surgery for choledochal cysts was performed using a hybrid approach that incorporated laparoscopy. The initial laparoscopic stage involved the dissection of the choledochal cyst, followed by the docking of the robotic system. (Fig. 1A, Fig. 1B) After docking, the robotic system facilitated the hepaticojejunostomy anastomosis. Although initially conducted entirely using the robotic system, it was decided to utilize the robot solely for the critical anastomosis phase due to concerns regarding the overall surgery duration due to excessive operation time of totally-robotic choledochal cyst excision. (Fig. 1C, Fig. 1D).

In both methods, postoperative management was the same regarding postoperative schedule of diet reintroduction, drain removal, laboratory tests, and ultrasound follow-up.

### Data collection

The collected data encompass all patients’ demographic information, the cyst’s type and size, and clinical outcomes. Clinical outcomes include operation time, estimated blood loss, irrigation of stones, postoperative hospital stay, postoperative complications, and follow-up days. The primary outcome was the presence or absence of postoperative complications, and the secondary outcome was satisfaction through a postoperative survey.

### Satisfaction survey questionnaire

An online survey was conducted to evaluate patient satisfaction with the surgical outcomes. The survey questionnaire included the Glasgow Children’s Benefit Inventory (GCBI) [14], the Korean version of the Gastrointestinal Quality of Life Index (GIQLI) [15], and 13 additional supplementary questions [11]. A question regarding sexual life in the GIQLI was excluded. All questions were translated into Korean and mailed to the patient’s parents. Two weeks after the initial mailing, the survey was resent. Out of 60 patients, 17 participated in the survey, with 15 participants from the laparoscopy group and 2 from the robot-assisted group. Due to the low number of participants in the robot-assisted group, a comparison between the two groups was not conducted.

### Statistical analyses

The collected data was analyzed using SAS OnDemand for Academics (SAS, Carry, NC). Continuous variables that did not meet the criteria for normality were compared using the Wilcoxon rank sum test. Categorical variables were evaluated using the chi-square test. All tests were two-sided; a P value ≤ 0.05 was considered statistically significant.

## Results

Characteristics and demographic data of the study population are described in Table 1. The male population was 28.3%, and the female was 71.7%. The average age of the patients was 58.97 months. The average body weight of the laparoscopy group (18.83 kg) was significantly lower than that of the robot-assisted group (26.07 kg). In the laparoscopy group, there was 1 patient with a chromosomal anomaly, 1 with a previous abdominal operation, and 4 with preoperative pancreatitis. The most common symptoms were abdominal pain (63.3%), followed by vomiting and jaundice (30.0%, 16.7%). A total of 7 patients (11.7%) were detected with prenatal ultrasonography. All patients underwent MRI scans before surgery. The cyst diameter identified through preoperative imaging had an average size of 2.46 cm. Stones identified through MRI before the operation accounted for 45.9%, and 8 patients underwent preoperative ERCP to remove the stones and sludge in the common channel.

**Table 1:** Patient demographics

	Total (N = 60)	Laparoscopy (N = 45)	Robot assisted (N = 15)	p
Sex				
Male	17 (28.3%)	12 (26.7%)	5 (33.3%)	0.7428
Female	43 (71.7%)	33 (73.3%)	10 (66.7%)	
Age at operation	58.97 [16.5;89.5]	49.38 [14.0;65.0]	87.73 [17.0;124.0]	0.074
Body weight	18.83 [22.8;9.8]	16.42 [9.4;18.2]	26.07 [38.2;10.2]	0.0388
Symptoms				
Abdominal pain	38 (63.3%)	27 (60.0%)	11 (73.3%)	0.3534
Vomiting	18 (30.0%)	15 (33.3%)	3 (20.0%)	0.5168
Jaundice	10 (16.7%)	8 (17.8%)	2 (13.3%)	1.0000
Prenatal sonography	7 (11.67%)	6 (13.3%)	1 (6.7%)	0.6678
Preoperative laboratory findings				
Total bilirubin	2.75 [0.7;3.6]	3.24 [0.6;4.8]	1.36 [0.7;1.9]	0.2268
Amylase	223.24 [54;197]	241.05 [54;197]	165.33 [56.5;214.5]	0.8852
Lipase	494.93 [20.5;416.5]	548.76 [22;526]	333.46 [15;216]	0.1254
Imaging				
Sono	51 (85.0%)	39 (86.7%)	12 (80.0%)	0.6777
CT	22 (36.7%)	19 (42.2%)	3 (20.0%)	0.1219
MRI	60 (100.0%)	45 (100.0%)	15 (100.0%)	0.0000
Todani classification				
1	51 (85.0%)	38 (84.4%)	13 (86.7%)	0.1594
2	1 (1.7%)	0 (0.0%)	1 (6.7%)	
4	8 (13.3%)	7 (15.6%)	1 (6.7%)	
Cyst diameter (cm)	2.46 [1.3;3.0]	2.69 [1.4; 3.2]	1.73 [1.1; 2.4]	0.0710
Stone/Sludge	28 (45.9%)	24 (53.3%)	4 (26.7%)	0.0730
Preoperative ERCP	8 (13.3%)	6 (13.3%)	2 (13.3%)	1.0000

Categorical variables are represented by frequency and percentage, while non-categorical variables are represented by mean and interquartile ranges

Operative outcomes are described in Table 2. The mean operation time for the total population was 213.92 min. For the robot-assisted group, the console time was 32.5 min. Differences between the groups in anastomosis site size, total hospital stay, and follow-up months were statistically significant. The average anastomosis site size was 9.38 mm for the laparoscopy group and 7.26 mm for the robot-assisted group. The average hospital stay was 14.64 days for the laparoscopy group and 11.13 days for the robot-assisted group. The average follow-up durations were 40.96 and 17.53 months, respectively. There were no statistically significant differences in complications between the groups; the robot-assisted group had no complications, while the laparoscopy group had 5 cases, including 2 of pancreatitis, 1 of A-loop syndrome, 1 of chyle ascites, and 1 of wound complication. Reoperations were performed for the treatment of afferent loop syndrome and wound complications.

**Table 2:** Operative outcomes

	Total (N = 60)	Laparoscopy (N = 45)	Robot-assisted (N = 15)	p
Operation time (min)	213.92 [180;237.5]	216.33 [185;245]	206.67 [175;225]^†^	0.1438
Anastomosis site size (mm)	8.86 [4.635;10.44]	9.38 [5.09;11.55]	7.26 [3.68;8.69]	0.0381
Estimated blood loss (mL)	65.27 [2.5;62.5]	49.31 [0;60]	113.13 [20;100]	0.2853
Complication rate	5 (8.3%)	5 (11.1%)	0 (0.0%)	0.1775
Average total hospital stay (days)	13.67 [8;15.5]	14.64 [9;16]	11.13 [8;12]	0.0224
Average postoperative stay (days)	9.38 [7;9]	9.91 [7;9]	7.8 [7;8]	0.3542
Follow-up duration (months)	35.1 [20;52]	40.96 [25;57]	17.53 [4;28]	0.0002

^†^ Docking time was 32.5 min [30;35]

Five cases of complications included two cases of pancreatitis, one of A-loop syndrome, one of chyle ascites, and one of wound complication

Categorical variables are represented by frequency and percentage, while non-categorical variables are represented by mean and interquartile ranges

Survey questionnaires and results are detailed in Tables 3 and 4. Of the 60 patients participating in this study group, 3 patients were excluded from the GCBI index because they did not meet the inclusion criteria. Of the 56 patients who met the inclusion criteria for GCBI, 14 patients (12 from the laparoscopy group and 2 from the robotics group) responded, representing a 25% response rate. Of the 60 patients eligible for the GIQLI, 17 patients (15 from the laparoscopy group and 2 from the robotics group) responded, reflecting a 28% response rate. Survey results indicated that overall satisfaction with the surgery was satisfactory. However, Table 3 reveals that patients were more susceptible to colds and required more medication post-surgery.

**Table 3:** GCBI survey results

	Total (N = 14)
GCBI Score	63.24 (78.125)
Has your child’s operation:*	
Made his/her overall life better or worse?	1.64 (2)
Affected the things he/she does?	1.64 (2)
Made his/her behavior better or worse?	1.57 (2)
Affected his/her prognosis and development?	1.29 (2)
Affected how lively he/she is during the day?	1.57 (2)
Affected how well he/she sleeps at night?	1.43 (2)
Affected his/her enjoyment of food?	1.29 (2)
Affected how self-conscious he/she is with other people?	1.50 (2)
Affected how well he/she gets on with the rest of the family?	1.43 (2)
Affected his/her ability to spend time and have fun?	1.14 (2)
Affected how embarrassed he/she is with other people?	1.07 (1.5)
Affected how easily distracted he/she has been?	1.07 (1.5)
Affected his/her learning?	1.07 (2)
Affected amount of time he/she has had to be off nursery, play group or school?	1.14 (2)
Affected his/her ability to concentrate on a task?	1.07 (1.5)
Affected how frustrated and irritable he/she is?	1.29 (2)
Affected how he/she feels about himself/herself?	1.07 (1.5)
Affected how happy and content he/she is?	1.64 (2)
Affected his/her confidence?	1.29 (2)
Affected his/her ability to care for himself/herself?	1.14 (2)
Affected his/her ability to enjoy leisure activities?	1.43 (2)
Affected how prone he/she is to catch colds?	0.79 (0)
Affected how often he/she needs to visit a doctor?	1.00 (1)
Affected how much medication he/she has needed?	0.79 (0.5)

^*^ Scores are given as mean (median)

Scores greater than 0 suggest improvement, scores of 0 indicate no change, and scores less than 0 suggest worsening

**Table 4:** GIQLI survey and supplementary question results

	Total (N = 17)
GIQLI Index	119.35 (123)
Symptoms (19 items)	68.00 (69)
Emotions (5 items)	15.24 (17)
Physical function (7 items)	23.12 (23)
Social function (3 items)	9.18 (10)
Medical treatment (1 item)	3.82 (4)
Supplementary questions*	
Postoperative pain	
Actual	4.12 (5)
Compared to expectation	2.29 (1)
Speed of return to normal activity	
Actual	4.18 (5)
Compared to expectation	3.82 (5)
Surgery incision scar	
Actual	3.94 (5)
Compared to expectation	3.71 (4)
Impact of surgery on parental life	
Actual	3.94 (5)
Compared to expectation	2.71 (3)
Burden of postoperative visits	
Actual outcomes	3.59 (4)
Compared to expectations	2.88 (3)
Overall satisfaction with surgery	4.29 (5)

Scores are presented as mean (median);

^*^ Scores for satisfaction with the actual outcome ranged from 1 (very dissatisfied) to 5 (very satisfied). Scores for outcome compared to expectations ranged from 1 (far less than expected) to 5 (far more than expected)

## Discussion

This study evaluated and compared the safety and efficacy of laparoscopic and robot-assisted procedures in pediatric choledochal cyst cases. The primary method for treating choledochal cysts is via excision using a Roux-en-Y hepaticojejunostomy with a laparoscopic approach [4, 16]. Benefits of the laparoscopic approach in choledochal cyst excision include improved cosmetic outcomes and reduced pain with early recovery compared to open surgery [17]. Conversely, robot-assisted surgery offers advantages such as minimal invasiveness, enhanced recovery and cosmetic outcomes, better surgical access due to its flexibility and maneuverability, and a three-dimensional view of the surgery site [3, 8]. However, there are limitations to robot-assisted surgery in pediatrics [18]. It is known that safety principles for older children and adolescents are similar to those for adult patients, but younger patients require careful consideration regarding positioning, anesthesia, and insufflation volumes [18]. Additionally, robotic instruments are only available in sizes 12.0 mm and 8.5 mm, significantly larger than the 3 mm instruments used in pediatric laparoscopic procedures. Scopes are only available in 12.0 mm and 8.5 mm sizes, which are too large for children under 5 kg [18]. Despite the limitations of the robotic surgical system, its short learning curve [19] and technical advantages at the anastomosis stage have been beneficial, and we started to adopt robot-assisted surgery at our center. Furthermore, following the first reported case of robot-assisted choledochal cyst resection by Woo et al. [7], similar studies conducted in various centers [20, 21] have confirmed that robot-assisted surgery is a viable option for treating choledochal cysts.

Our study is a retrospective study in a single center, analyzing data from a single surgeon. There were some noticeable results from our analyzed data. Our data indicates that robot-assisted surgery in choledochal cyst excision offers benefits in treating children with small cysts with small anastomosis sites. Although not statistically significant, patients who underwent robot-assisted surgery (88 months) were older than those in the laparoscopic group (49 months). In the robot group, we successfully conducted excisions in neonates, including one case involving a 1-month-old child and another involving a 4-month-old child, both without complications. Additionally, the diameter of the cyst was smaller in the robot-assisted group (1.73 cm) compared to the laparoscopic group (2.69 cm). The anastomosis site, which is the common hepatic duct, was also smaller. The laparoscopic group had an average common hepatic duct size of 9.38 mm, while the robot-assisted group showed an average of 7.26 mm. After several cases of complete robot surgeries, we utilized the robotic system only at the anastomosis stage. The flexibility and maneuverability of the robotic surgery system proved effective during the anastomosis stage of the surgery, even at a much smaller site. Better ergonomics of the robotic surgical system are reported to enable more precise suturing, as noted in other literature [3, 8].

Furthermore, our findings indicated no statistically significant difference in operation times between the laparoscopic group (216.33 min) and the robotic group (206.67 min), contradicting previous studies [20, 22]. Xie et al. noted that laparoscopic procedures required more time due to the technical difficulties associated with limited abdominal space and the inherent challenges of laparoscopic techniques [20]. Zhang et al. observed that robotic surgeries took longer due to the time needed for docking and instrument replacement [22]. Our center reported similar operation times for both groups, likely due to the exclusive use of the robotic system during the anastomosis stage and our proficiency with both robotic and laparoscopic surgeries. This suggests that robot-assisted surgery is viable in terms of operational duration.

Concerning complications, both groups showed similarly low rates, with the robot-assisted group experiencing no complications, while the laparoscopic group had 5 cases (11.1%). Regarding complications, reoperations were performed for patients with afferent loop syndrome and chyle ascites. Nonetheless, complications may increase in the future with more patients undergoing robot-assisted surgery and more extended follow-up periods. Kim’s retrospective study compared robot-assisted to open procedures and found no differences in complication rates [23].

The total hospitalization duration was shorter for patients in the robot-assisted group. Since there was no difference in postoperative days between the groups, this suggests that the laparoscopic group required more time for preoperative care. Although the mean age of the laparoscopic group was younger and although not statistically significant, it implies that preparation for surgery took longer in this group.

In the satisfaction survey for the Robot-assisted surgery group, the response rate was notably low, with only 2 out of 15 patients participating. Unlike our approach, which exclusively utilized an online survey, previous studies employed methods such as departmental billing systems, telephone surveys, and patient diaries, resulting in much higher response rates [11, 24, 25]. Additionally, the Robot-assisted surgery group reported significantly lower satisfaction scores overall, which contradicts other studies that reported higher satisfaction with robot-assisted techniques [11–13, 26]. To validate these results and compare the two groups, future studies should include more participants.

However, this study has some limitations. First, there is potential bias because the choice of surgical method was made by the patient’s legal guardian, not through a randomized process. Moreover, the 3:1 ratio of laparoscopic to robot-assisted cases may introduce additional bias. Equalizing the number of cases across groups and increasing the sample size could enhance the accuracy of the results.

Robot-assisted surgery represents a viable and effective method for treating pediatric choledochal cysts, yielding comparable outcomes to laparoscopic procedures. The hybrid robot-assisted technique allowed for equally lengthy surgeries compared to laparoscopic approaches and facilitated more precise excisions in smaller anastomosis areas. Additionally, robot-assisted surgery will likely become more widespread and increasingly adopted by various organizations due to its advantages.
